# Knowledge, attitudes and practices of nurses and pharmacists towards adverse drug reaction reporting in the South African private hospital sector

**DOI:** 10.4102/hsag.v23i0.1064

**Published:** 2018-11-12

**Authors:** Sophia Bogolubova, Neelaveni Padayachee, Natalie Schellack

**Affiliations:** 1 Department of Pharmacy and Pharmacology, University of the Witwatersrand, South Africa; 2 School of Pharmacy, Sefako Makgatho Health Sciences University, South Africa

## Abstract

**Background:**

Pharmacovigilance (PV) is an important tool in monitoring the quality, efficacy and safety of medicines, with spontaneous reporting being the mainstay system of reporting adverse drug reactions (ADRs) worldwide. ADRs are largely underreported in South Africa.

**Aim:**

This study aimed to evaluate the knowledge, attitudes and practices of pharmacists and nurses in the private hospital sector towards ADR reporting.

**Setting:**

This study was conducted in six private hospitals and clinics within a single hospital group in Johannesburg, South Africa.

**Method:**

A cross-sectional survey was conducted amongst healthcare professionals using a structured multiple-choice questionnaire containing 20 close-ended questions. Both electronic and paper questionnaires were used to obtain as many responses as possible.

**Results:**

A total of 233 healthcare professionals participated in the study. Of these, 78.5% were registered nurses and 21.5% were hospital pharmacists. Although three-quarters of participants believed ADR reporting to be important, most had received no previous PV training (76.2%) and did not know how to report an ADR (54.5%). The majority of participants (87.1%) believed that all ADRs should be reported, with 75.5% of participants believing they would report all ADRs they encountered in the future provided they had sufficient training and knowledge. The major factors discouraging participants from reporting was a lack of awareness with respect to the process of reporting as well as a lack of access to the ADR reporting form.

**Conclusion:**

The majority of participants require further training regarding ADR reporting. Although the knowledge of most participants was acceptable, the transition into practice needs to be improved.

## Introduction

### Pharmacovigilance in South Africa

Pharmacovigilance (PV) in Africa is still largely considered to be in its infancy (Olsson, Pal & Dodoo 2015). Improving access to life-saving medicines took precedence over PV in low- to middle-income countries, especially in most African countries, before the availability of global funding improved accessibility to these medicines. The emphasis on access to medicines at the expense of PV, however, increased the risk of treatment-related adverse effects, especially in communities with limited education and few trained healthcare professionals (HCPs) (Olsson et al. 2015). With the emergence of a larger middle class in recent years that is able to pay for their medications, national development programmes have shifted their focus away from emphasising access of medicines towards the establishment of safety and quality surveillance systems for these medicines (Ampadu et al. 2016).

Underreporting of adverse drug reactions (ADRs) is considered to be a global issue. In South Africa, where PV and other regulatory aspects of medicine use are not yet fully developed, ADR reporting rates are still very low considering how long PV has been functioning. South Africa has submitted a total of 28 609 reports to VigiBase® since an official PV system began functioning in 1992 (Ampadu et al. 2016). This amounts to approximately 27 reports per million per year. Considering the high number of people living with human immunodeficiency virus (HIV), tuberculosis (TB) and other communicable and non-communicable diseases in South Africa, the number of reports submitted to VigiBase® is expected to be higher (Stats SA 2016; WHO 2016).

The private healthcare sector in South Africa is a seldom studied field of healthcare. Most studies conducted in the country tend to focus on public sector facilities and patients (Ganesan et al. 2016; Isah et al. 2012; Joubert & Naidoo 2016; Mouton et al. 2015, 2016; Roux 2014; Ruud, Srinivas & Toverud 2010; Strengthening Pharmaceutical Systems Program 2011; Suleman 2010). While the public healthcare sector serves the majority of the population, studies conducted in this sector are therefore useful when investigating public health issues. However, the private sector is associated with a greater supply and availability of medicines (i.e. there is a wider variety of drugs available, greater range of generic medicines available, as well as expensive specialised drugs that are not available in the public sector). The private sector in South Africa provides primary healthcare (PHC) services for approximately 28% – 37% of the population because of the private–public sector relationship, but in reality this figure is estimated to be at approximately 17% (Econex 2013).

*Guideline 2.3.3 Reporting of Post-Marketing ADRs to Human Medicinal Products in South Africa* (December 2015), published by the Medicines Control Council (MCC), places the responsibility for ADR reporting largely on the holders of the certificate of registration of medicines. It makes no provision to place responsibility on HCPs (such as doctors, nurses or pharmacists) to report ADRs, despite these professionals being the most likely point of first contact. Although HCPs are encouraged and professionally obliged to report ADRs, how much information is gathered, and consequently reported, is dependent on the awareness and assertiveness of the HCP (Pimpalkhute et al. 2012).

Currently, there are a number of PV systems in South Africa. PV is a mandated function of the MCC and they are responsible for the regulatory aspects of PV, that is, signal detection, ensuring provision of safe, effective and quality medicines, post-marketing surveillance, instituting appropriate remedial action and establishing the risk–benefit profile of all registered medicinal products (Maigetter et al. 2015). The other PV system is that of the National Pharmacovigilance Center (NPC), which is responsible for coordinating PV in the public health programmes, particularly at PHC level. This decentralisation aims to increase the interest of PHC workers with respect to medicines and medicine safety.

In addition to the MCC and the NPC, there are a number of separate entities such as the Adverse Event Following Immunization System, the Operational Plan for Comprehensive HIV/AIDS Care, as well as non-governmental organisations such as the Wits Health Consortium, that have developed their own PV programmes that do not always feed into the national MCC system (Essack et al. 2011). Although the MCC is responsible for the management of these systems, there is no formal relationship between the MCC and other PV centres, nor is there any system of peer review of the responsible units (Essack et al. 2011).

The current PV framework in South Africa is complex and convoluted as a result of the many possible arms of reporting, altering the direction of reporting and creating uncertainty for HCPs. Although a PV framework exists for reporting, the communication on where reports should go is unclear. The trend is that data are often not fed to a national system, or are not fed centrally, which is evident from fewer generated reports (Maigetter et al. 2015). Without a full understanding of the flow of reporting, practitioners may fail to see why reporting ADRs is worth the time invested. The lack of awareness regarding the process of reporting to a national ADR reporting system is cited as a common barrier to reporting (Suyagh, Farah & Farha 2015).

### Incidence of adverse drug reactions

ADRs affect a number of patients worldwide, irrespective of age, gender, location or occupation, and can affect patients with varying magnitudes leading to morbidity and mortality (Pirmohamed et al. 2004). Lazarou, Pomeranz and Corey (1998) estimated that ADRs could be considered to be the fourth to sixth leading cause of death in the USA, with the incidence having remained stable over the previous 30-year period. ADRs then became a cause of death ahead of diseases such as pneumonia and diabetes (Lazarou et al. 1998). Additionally, a meta-analysis conducted by Wiffen (2002) of 69 prospective and retrospective studies worldwide involving 419 000 patients concluded that ADRs were responsible for approximately 6.7% of all hospitalisations. The use of self-medication, fake and adulterated medicines, as well as traditional and herbal therapies, increases the burden of ADRs in developing countries such as South Africa (Suleman 2010).

A recent study conducted in four hospitals in the public sector in South Africa by Mouton et al. (2016) found that 1 in 12 hospital admissions were because of an ADR. Of these patients, 58% were taking more than five drugs at time of admission (ranging from 1 to 17 drugs) and 39% of admitted patients were HIV positive (Mouton et al. 2016). No similar studies have thus far been conducted in the private sector, highlighting the necessity for greater adherence to PV practices. South African patients tend to provide the perfect landscape for ADRs as a result of the cocktail of medications prescribed because of the high incidence of HIV, TB and non-communicable diseases (Mehta 2011). Additionally, herbal and traditional medicines are a popular choice for many South Africans because of their low costs and free availability. The market for these medicines is estimated at approximately R3 billion, with at least 27 million people consuming herbal or traditional medicines annually (Essack et al. 2011; BMI 2010).

### Underreporting

The reasons for low reporting of ADRs by HCPs have been well researched. Lopez-Gonzalez, Herdeiro and Figueiras (2009) released a systematic review mentioning ignorance (95%), diffidence (72%), lethargy (77%), indifference and insecurity (67%) and complacency (47%) as the primary reasons for underreporting. The paperwork involved with such reporting seems to discourage the desire to produce data of any sort, especially because those responsible for reporting perceive the data as irrelevant to their immediate clinical needs (Ruud et al. 2010). This could also pose a great challenge to inexperienced HCPs, who may lack the sound clinical judgement needed to determine a causal relationship between an adverse or unexpected event and a drug (Suleman 2010).

Factors relating to processes for reporting, such as inadequate feedback, long forms and insufficient time to report, are often identified as major barriers to reporting (Van Hunsel et al. 2010). A pharmacist interviewed in a study conducted by Ruud and colleagues aptly stated:

you report in a vacuum. You give it to somebody and you never hear again. And it’s nice to get feedback, from whoever who are collecting these ADRs to say, look, this is what we’re looking for, this is not what we’re looking for. (Ruud et al. 2010:345–353)

The statement is supported by a previous study conducted by Evans et al. (2006), in which 58% of HCPs cited a lack of feedback as a self-perceived barrier to reporting (Van Hunsel et al. 2010). Within the South African context, pharmacists in a study conducted by Joubert and Naidoo (2016) in 2016 felt PV centres were inaccessible with little to no personal contact. Other self-perceived barriers noted by HCPs in the study conducted by Evans et al. included the form taking too long to complete, a lack of time, not wanting to take responsibility for the report and believing that the report would not make any difference (Van Hunsel et al. 2010).

In order to improve ADR reporting rates in South Africa, an analysis of the current state of PV activity needs to take place. It is important to understand the reasons why HCPs are not making PV a priority activity in the management and treatment of their patients in order to determine the best methods for improvement.

### Aims and objectives

The study aimed to evaluate the knowledge, attitudes and practices of pharmacists and nurses in the private hospital sector towards ADR reporting. The objectives included, but were not limited to, establishing factors that contributed to differences in both knowledge and attitudes towards ADR reporting, as well as exploring trends that interfered with effective ADR reporting.

## Methodology

### Study design

The study design involved a cross-sectional, observational, questionnaire-based survey of registered nurses and hospital pharmacists working in the private sector within a single hospital group.

### Study population

The research was conducted in six private hospitals and clinics within a single hospital group in Johannesburg, South Africa. The hospitals were selected using a purposive sampling method as each hospital offers a variety of wards and specialties (i.e. maternity, paediatrics, oncology, intensive care unit, neurology, psychiatry, gynaecology, orthopaedics, neonatology and surgery) and provides both inpatient and outpatient facilities. It was therefore possible to include a study population with varying training and specialities, in order to obtain a broader spectrum of results.

Inclusion criteria for participants were as follows:

- registered nurse or hospital pharmacist employed at the facility (locum and agency staff included)
- willingness to participate (signed informed consent and/or completed questionnaire).

Exclusion criteria were as follows:

- non-willingness to participate
- enrolled nurses (i.e. had not yet completed their qualification)
- support pharmacy staff (i.e. pharmacist assistants, pharmacist interns and pharmacy students).

According to the published findings of a study conducted by Econex on behalf of the South African Private Practitioners Forum and HealthMan (Pty) Ltd, entitled *The South African Private Healthcare Sector: Role and Contribution to the Economy*, there were an estimated 77 569 nurses and 2984 pharmacists working within the South African private sector, using 2013 statistics (Econex 2013). Therefore, a sample size of 382 was calculated, using a confidence interval of 5 and a confidence level (*Z*) of 95%.

### Questionnaire development

A self-administered questionnaire was used as the primary data collection tool. It had been adapted from similar studies investigating the knowledge, attitudes and practices of ADR reporting amongst HCPs and modified to suit a South African private sector setting (Gupta & Udupa 2011; Jose et al. 2014; Kiran et al. 2014; Rajiah, Maharajan & Nair 2016; Van Hunsel et al. 2010).

The questionnaire contained 20 close-ended questions, with four of these providing an opportunity for an open-ended answer in the form of the option ‘Other – please specify’.

The questionnaire was designed to capture the following information:

- **Participant information and demographic (four questions)**. This included profession, gender, age and years of experience. The data was used to determine whether differences in these variables contribute to differences in knowledge, attitude or practice.
- **Background knowledge of the participant with regard to ADR reporting (six questions)**. This included previous training received, knowledge of ADR reporting form, where the ADR reporting form is located and where completed reports should be submitted. The data was used to determine the baseline knowledge of each participant towards ADR reporting and the ADR reporting process. This also aided in determining the level of previous exposure of each participant to ADRs and/or ADR reporting.
- **Participant perceptions towards ADR reporting (five questions)**. This included each participant’s perceived importance of ADR reporting in general, important or unimportant reasons for ADR reporting, factors that encouraged or discouraged reporting of ADRs and which kind of ADRs the participant thought should be reported. The data was used to determine the general attitudes of the participants towards ADR reporting and attempted to identify factors outside of the participants’ knowledge that may contribute to low reporting rates.
- **ADR reporting practices of participants (five questions)**. This included whether the participant had come across an ADR previously, whether they have previously reported an ADR, the likely circumstances under which the participant would submit an ADR report and which medical professional the participant deemed responsible for submitting ADR reports. This was to gain an understanding of the current ADR reporting practices of each participant in order to determine how it can be improved.

### Questionnaire distribution

The questionnaire was distributed to potential participants during the period June to December 2016. The questionnaire was distributed in two ways: electronically via e-mail to participants with regular computer and e-mail access at the workplace and manually via hard copy to participants without regular access to a computer or e-mail at their workplace. A list of e-mail addresses of potential participants was obtained from the pharmacy and nursing managers of each respective hospital. A total of 83 potential participants were identified for electronic questionnaire distribution, which included pharmacists, locum pharmacists and registered nurses. The registered nurses identified for electronic distribution all held senior or managerial positions. All other nurses (between 20 and 110, depending on the individual hospital) did not have regular access to computer or e-mail at their workplace and were thus considered for manual questionnaire distribution. Hard-copy questionnaires (including information sheet and informed consent document) were distributed to all potential participants after holding a brief meeting with the staff of every unit or department in each identified hospital. The meeting provided each potential participant with the same information contained in the information sheet and informed consent document. An excess number of questionnaires were provided to the manager of each unit or department for distribution to night staff and staff who were on leave or were otherwise absent. Participants were provided with a period of 1 month to complete the questionnaire. A total of 360 questionnaires were involved in the manual distribution. Therefore, a total of 443 questionnaires were distributed (83 electronic and 360 hard copies).

### Data analysis

Data were captured into Google Forms™ and then exported into Microsoft Excel 2016™. Descriptive data analysis was conducted using Microsoft Excel 2016. Each variable category was coded with a number for ease of analysis. Pearson chi-squares were used for a test of association, as well as cross-tabulation methods for bivariate analysis. Results are presented by means of percentages and/or graphs depending on their appropriateness to the variable in question. The relationship between different variables (e.g. age of respondent vs previous exposure to ADRs) was determined using a Pearson chi-square at *p* < 0.05. Frequency analysis was also employed to assess differences in attitudes, knowledge and practices.

### Ethical considerations

Ethical clearance to conduct this study was obtained from the University of the Witwatersrand Human Research Ethics Committee (Ethics No. M160238). Clearance was also obtained from the hospital group involved in the study on condition of confidentiality (approval number 20160620-01). Each potential participant was provided with an information sheet detailing the nature of the study and any benefits or risks to choosing to participate. All potential participants were informed that should they decide to withdraw or not complete the study, no repercussions, consequences or penalties would be applied. Informed consent was obtained from each participant prior to completion of the questionnaire, either electronically or manually.

## Results and discussion

### Demographics

A total of 233 HCPs completed the questionnaire. Thus, the response rate was 52.59%. The majority of participants were registered nurses, and approximately a fifth were pharmacists. Table 1 elaborates on the demographics of the participants.

**TABLE 1:** Frequency distribution of demographic characteristics (*n* = 233).

Demographic characteristics	Frequency (*n*)	Percentage (%)
**Profession**		
Registered nurse	183	78.5
Hospital pharmacist	50	21.5
**Total**	**233**	**100.0**
**Gender**		
Male	23	9.9
Female	210	90.1
**Total**	**233**	**100.0**
**Age**		
18–29 years old	49	21.0
30–39 years old	71	30.5
40–49 years old	61	26.2
50 years and older	52	22.3
**Total**	**233**	**100.0**
**Years of experience**		
Less than 1 year	17	7.3
1–5 years	42	18.0
5–10 years	63	27.0
Longer than 10 years	111	47.6
**Total**	**233**	**100.0**

### Knowledge

Thirty-two per cent of participants had previously seen the ADR reporting form, while over three-quarters (76%) did not know where it was located. Of those who had previously seen the form, 60% knew where it could be located, while a further 6.3% of participants who had not seen the form before knew where it could be found.

Over three-quarters of the participants (76.2%) had never received any type of PV or ADR reporting training. Of those that had, 17 were pharmacists and 37 were nurses. Pharmacists were more likely than nurses to have received training (*p* = 0.040685). However, to have three-quarters of participants in this study (179 of 233 respondents) having received no PV training during their careers can be viewed as problematic. The population of patients seen in hospitals is often vulnerable and prone to the development of ADRs because of the polypharmacy often used (Mouton et al. 2015). By virtue of the type of patient presenting in a hospital, a rudimentary understanding of PV might be beneficial to these patients and to the general community as a whole.

Approximately 30% of participants thought that they knew the process to follow when completing and submitting an ADR report. Over half (54.5%) did not know the procedure. Those who had received previous PV training were more likely to understand the ADR reporting procedure (*p* < 0.001). This finding is supported by numerous other sources that conclude that PV training increases the likelihood that HCPs will participate in PV activities such as ADR reporting (Maigetter et al. 2015).

Despite the form being entitled the ‘Medicines Control Council Adverse Drug Reaction Reporting Form’, 26.6% of participants stated that reports should be submitted to the MCC. This finding is supported by numerous studies where HCPs are either completely unaware of a national PV centre or authority, or are aware of its existence but not of its location, purpose or function (BMI 2010; Evans et al. 2006; Isah et al. 2012; Mouton et al. 2016; Roux 2014). Almost half (46.8%) did not know where the form should be submitted, while the other responses were spread between pharmacy manager (39.1%), nursing manager (23.6%), National Adverse Drug Event Monitoring Centre (16.7%), head office (8.6%) and hospital manager (3.0%).

When respondents were asked whether they believed their respective hospital submitted sufficient and/or appropriate ADR reports, 78.7% respondents said they did not know. A further 4.3% of respondents indicated that they thought their hospital submitted appropriate ADR reports while 17.6% said they did not believe so. This indicates that staff are largely not informed in such matters. Additionally this might indicate that there are insufficient processes in place for the handling of ADR reports.

### Attitude

In total, three-quarters of respondents (75.96%) thought that reporting ADRs was very important (Table 2). Opinions between nurses and pharmacists were similar, with the exception of three nurses believing ADR reporting to be not important.

**TABLE 2:** Importance placed on adverse drug reaction reporting: Nurses versus pharmacists.

Importance placed	Nurses		Pharmacists		Total	
		
	Frequency (*n*)	Percentage (%)	Frequency (*n*)	Percentage (%)	Frequency (*n*)	Percentage (%)
Very important	133	72.60	44	88.0	177	75.96
Important	47	25.68	6	22.0	53	22.75
Not important	3	1.64	0	0.0	3	1.28
**Total**	**183**	**100.00**	**50**	**100.00**	**233**	**100.00**

More specific statements regarding the importance of ADR reporting showed similar opinions between nurses and pharmacists: ‘I think it is important to report ADRs to identify new ADRs’ (80.33% vs 86.00%); ‘I think it is important to report ADRs to share information with colleagues’ (68.85% vs 70.00%); ‘I think it is important to report ADRs to help establish the safety of new drugs’ (81.42% vs 88.00%); ‘I think it is important to report ADRs to measure the incidence or frequency of ADRs’ (70.49% vs 80.00%); ‘I think it is important to report ADRs because it is a legal requirement’ (60.11% vs 56.00%).

When asked which type of ADRs should be reported, most respondents thought that ADRs to all types of drugs should be reported. Only 22.3% believed that ADRs to new drugs should be reported (Table 3). This viewpoint can be considered as a problematic attitude, as long-term harm caused by new drugs is often not known when they are first marketed. Even though post-marketing surveillance is compulsory for pharmaceutical manufacturers as per Guideline 2.3.3, mentioned previously, HCPs should still consider ADR observation and reporting to be a priority activity in the management of new drugs, considering that they are often the most likely point of first contact with ADRs. Thirty participants thought that ADRs to herbal, natural or traditional medicines should be reported, although establishing causal relationships may be difficult as the contents of traditional and herbal medicines are often unknown, and in some instances contain potentially harmful ingredients (Isah et al. 2012).

**TABLE 3:** Type of adverse drug reactions that should be reported: Nurses versus pharmacists.

Type of ADR	Nurses (*n*)	Pharmacists (*n*)	Total	

			Frequency (*n*)	Percentage (%)
None	1	0	1	0.4
All ADRs	169	34	203	87.1
All serious ADRs (causing death or serious injury)	47	27	74	31.8
ADRs to medical devices (such as pacemakers, prosthetics, etc.)	24	19	43	18.5
ADRs to new drugs	28	24	52	22.3
ADRs to herbal, natural or traditional medicines	18	12	30	12.9

ADR, adverse drug reaction.

Two respondents (both pharmacists) provided the following comments in response to the questions regarding which ADRs should be reported:

‘All ADRs necessitating change of therapy.’ (Pharmacist, female, practicing for longer than 10 years)

‘ADRs not specified on package insert.’ (Pharmacist, female, practicing for longer than 10 years)

**FIGURE 1:**
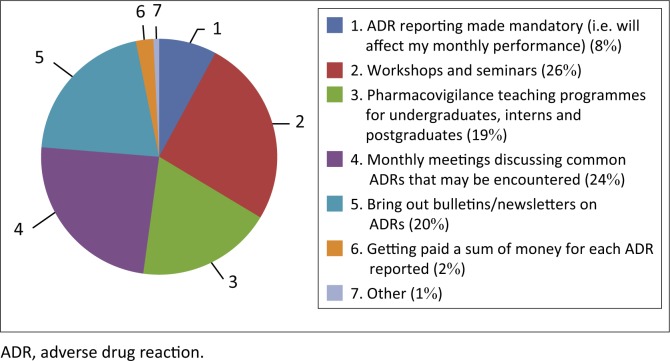
Suggestions on how the culture of reporting can be improved.

When asked for suggestions on how to improve the ADR reporting culture at their respective hospitals, most respondents seemed to prefer in-house methods to increase training: workshops and seminars (55.8%); monthly meetings discussing common ADRs that may be encountered (52.4%); bring out bulletins or newsletters on ADRs (44.6%). Four participants provided additional comments regarding how to improve ADR reporting at their hospitals (Figure 1):

‘When is a reaction an ADR. How to identify and determine when to report.’ (Pharmacist, male, practicing for longer than 10 years)

‘Online reporting.’ (Pharmacist, female, practicing for 1–5 years)

‘Electronic submission process with instant feedback on status of the ADR reported.’ (Nurse, female, practicing for longer than 10 years)

‘Access of ADR forms.’ (Pharmacist, female, practicing for 1–5 years)

These comments support findings obtained internationally that varying degrees of unfamiliarity with the reporting process remains one of the biggest hurdles to efficient reporting (Ganesan et al. 2016; Joubert & Naidoo 2016).

### Practice

Only 18.9% of participants (i.e. *n* = 44) stated that they had previously reported an ADR. Of these, 13 respondents were pharmacists and 31 were nurses. Participants who had received PV training in the past were also more likely to have reported an ADR in the past (*p* = 0.00048). When the participants were asked whether they had previously encountered an ADR and failed to report it, 13.7% indicated ‘yes’, while two-thirds of the participants indicated ‘no’. The remaining 22.3% stated that they *‘*didn’t know’, that is, they were not sure if they had ever encountered an ADR.

Older participants (aged 40 and older) were more likely than younger participants (aged 40 and younger) to have reported an ADR in the past (21.48% vs 16.07%) (*p* = 0.291, therefore not statistically significant) and were more likely to know the ADR reporting process (35.54% vs 23.21%) (*p* = 0.039, therefore statistically significant). This corresponds to a similar study by Evans et al. (2006) where senior nurses had a higher degree of involvement in the ADR reporting process than their junior counterparts (Evans et al. 2006).

**FIGURE 2:**
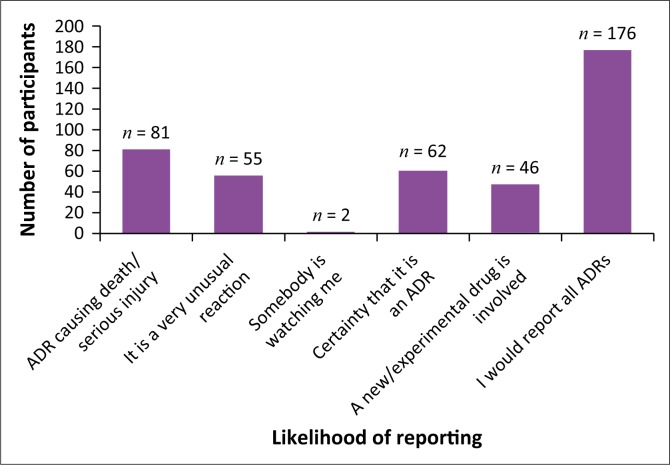
Likelihood that participants would report an adverse drug reaction (ADR) (*n* = 233).

The majority of participants (75.5%) stated that they would most likely report all ADRs they encountered (Figure 2). Therefore, more effort to train these HCPs to report ADRs would be beneficial. If a similar intention to report all ADRs exists for the majority of HCPs, the process of reporting needs to be streamlined and made more efficient, as well as providing more integrated and intensive training regarding identifying and detecting ADRs.

There were a number of factors that participants stated discouraged them from reporting ADRs. The frequency of these factors varied greatly, as summarised below in Table 4. It is possible to surmise that the three biggest factors that prevent HCPs from reporting ADRs are not knowing how to report, not knowing where to report and a lack of access to ADR forms.

**TABLE 4:** Factors that might discourage healthcare professionals from reporting adverse drug reactions (ADR).

Factors	Frequency (*n*)	Percentage (%)
Do not know how to report	108	46.4
Do not know where to report	81	34.8
Did not think it was important to report	18	7.7
Managing the patient was more important than reporting the ADR	27	11.6
Lack of access to ADR reporting form	80	34.3
Patient confidentiality might be breached	10	4.3
Legal liability issues	6	2.6
The form is too long	19	8.2
I don’t receive any feedback once the form has been sent	32	13.7
Other (comments provided by participants as summarised below)	17	7.3

Most of the additional comments provided by participants exemplified the fact that HCPs are simply not educated and/or trained enough in the identification of an ADR. Admittedly, many ADRs might be quite subtle and difficult to distinguish from an actual clinical disease state. Sometimes it might be impossible to directly identify an ADR. In this respect, it is vital that all HCPs work together in order to utilise the expertise of all fields, that is, the clinical expertise of doctors, the patient knowledge and care expertise of nurses and the pharmaceutical knowledge of pharmacists. In addition to the predefined factors, a number of comments were provided by the participants (summarised as follows):

‘Certainty as adverse reactions could be as a result of other factors not the treatment. It is difficult to know when a reaction is an ADR versus from some other cause.’ (Pharmacist, male, practicing for longer than 10 years)

‘Have not been in that situation yet.’ (Pharmacist, female, practicing for 1–5 years)

‘Don’t know how to tell if ADR.’ (Nurse, female, practicing for longer than 10 years)

‘None because I have no experience with doing such.’ (Nurse, female, practicing for 5–10 years)

‘Lack of training regarding reporting the ADR.’ (Nurse, female, practicing for longer than 10 years)

‘This is my first time seeing the ADR form.’ (Nurse, female, practicing for 5–10 years)

‘Not knowing what an ADR is.’ (Nurse, female, practicing for longer than 10 years)

‘No internal process for ADR.’ (Nurse, female, practicing for longer than 10 years)

‘Not sure if it might be an allergic reaction that the patient did not know.’ (Nurse, female, practicing for 5–10 years)

**FIGURE 3:**
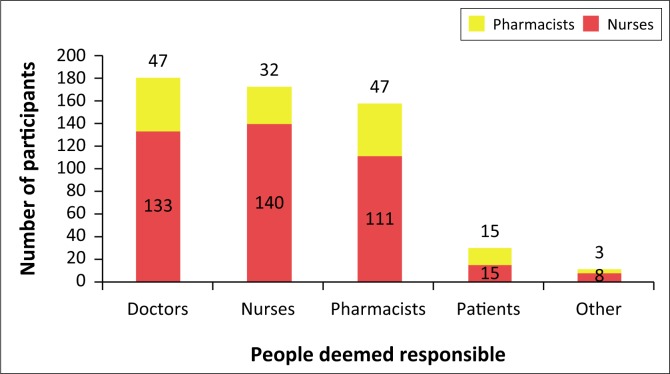
People deemed responsible by the participants for reporting adverse drug reactions (*n* = 233).

Participants also had an opportunity to indicate who they believe should be responsible for reporting ADRs (Figure 3). A small portion of participants (4.7%), in addition to marking the boxes for doctors, nurses and pharmacists, marked the box for ‘Other’ to state that they believe all HCPs should be responsible for reporting ADRs. This finding is supported by a similar study where only 8.8% of pharmacists correctly believed that all HCPs were role players in the ADR reporting process (Jose et al. 2014). One participant aptly stated, ‘all the above professionals because they prescribe, dispense and administer these drugs to patients’.

Only 12.87% of participants (15 pharmacists and 15 nurses) in this study thought that patients should be responsible for reporting ADRs. In practical terms, patients are not always suitably qualified to report ADRs because of a lack of knowledge or awareness, and therefore better communication between patients and HCPs needs to be encouraged. While HCPs have the main responsibility of reporting ADRs, patients have been permitted and should be encouraged to report ADRs in countries such as South Africa in order to increase reporting rates (Joubert & Naidoo 2016; Raza & Jamal 2015).

### Strengths and limitations

The study design is considered to be a strength in that a number of different hospitals were used in order to include participants from differing specialties in varying settings so as to obtain a broad spectrum of results. A major limitation was that only one hospital group was utilised. It might be possible that other hospital groups in South Africa have different knowledge, attitudes and practices of ADR reporting. Another limitation is that doctors were unfortunately excluded from this study because of a lack of willingness to participate during piloting.

### Recommendations

Based on some of the more significant findings of this study (e.g. participants largely not knowing how to report, or not knowing what an ADR is), it would be beneficial to conduct an intervention type study to determine whether knowledge, attitudes and practices would change after implementing an intervention such as a workshop or training seminar. It might also be beneficial to conduct a similar type of study on a larger scale in order to obtain a more accurate representation of the current knowledge, attitudes and practices in the private healthcare sector.

## Conclusion

The knowledge of the participants of this study with respect to ADR reporting is inadequate. Regardless of their profession, the participants involved in this study did not provide satisfactory answers regarding the ADR reporting form and the processes involved with it, including who should be responsible for reporting. Largely it would appear that the primary reason for participants not knowing where the form must be submitted was that they had simply never seen the form before. However, the overall knowledge of participants regarding ADR reporting could be considered as acceptable considering that only approximately a quarter of participants had ever received any previous PV training.

The attitude of participants to ADR reporting was overall quite positive. Most participants believed ADR reporting to be an important function of their job, with many of these agreeing that it was a professional obligation. A small cause for concern was the type of drugs participants believed should be reported, with only a small percentage believing ADRs to new drugs should be reported. Regardless, most respondents agreed that ADRs to all types of drugs should be reported.

Participants provided useful suggestions as to how to increase the culture of reporting at their respective hospitals. Considering that many had received no previous PV training, a large number of participants suggested in-house methods of training such as workshops and seminars in order to familiarise themselves with both the identification of common ADRs as well as the process of ADR reporting.

Although overall attitudes towards ADR reporting are quite positive, the overall knowledge is largely inadequate and the transition into practice needs to be improved. Only a small percentage of participants had previously reported an ADR before. The three biggest factors that prevent HCPs from reporting ADRs are not knowing how to report, not knowing where to report and a lack of access to ADR forms. In the greater scheme of things, these are minor issues that can be easily rectified. Most of the additional comments provided by participants exemplified the fact that HCPs are simply not educated and/or trained enough in the identification of an ADR.

In reality, improving PV in South Africa is an effort that must be based at national level. However, while those at national levels are slowly implementing improvements and changes, hospitals and clinics with the ability and resources to implement their own improvements should be encouraged to do so. Generally, attitudes of ‘one report will not make a difference’ need to be discouraged. Even if every private hospital in the country submits one report, it will make a difference in the certainty of the safety profile of a particular drug. Particularly in the private sector where there is a massive expenditure per annum on medicines, it can only benefit the population to increase the reporting rate.
